# E-cigarette, or vaping, product use-associated lung injury: a review

**DOI:** 10.1186/s41479-020-00075-2

**Published:** 2020-10-25

**Authors:** Samuel H. Belok, Raj Parikh, John Bernardo, Hasmeena Kathuria

**Affiliations:** Section of Pulmonary and Critical Care Medicine, Boston University School of Medicine, 72 E Concord St., R-304, Boston, MA 02118 USA

**Keywords:** EVALI, Vaping, Inhalational injury, E-cigarettes

## Abstract

**Background:**

E-cigarette, or Vaping, Product Use-Associated Lung Injury (EVALI) is a disease entity related to the use of battery-operated or superheating devices that create an aerosolized form of nicotine and tetrahydrocannabinol (THC) and/or other substances for inhalation.

**Methods:**

We performed a literature review to document epidemiology, pathogenesis and risk factors, diagnosis, clinical presentation, evaluation and management of EVALI.

**Results:**

In the summer of 2019, an outbreak of EVALI cases brought this disease entity into the national spotlight. Since being recognized as a serious pulmonary disease with public health implications, more than 2600 cases have been reported to CDC with 68 deaths as of February 2020. The pathophysiology of EVALI remains unknown. Substances such as Vitamin E acetate have been implicated as a possible causes of lung injury. The CDC has established case definitions of “confirmed EVALI” cases to help guide identification of the disease and assist in surveillance. While clinical judgement by healthcare providers is imperative in the identification of EVALI cases, the heterogeneous presentations of EVALI make this difficult as well. Ultimately most investigative studies should be aimed at ruling out other disease processes that can present similarly. Treatment is centered around removing the offending substance and providing supportive care.

**Conclusions:**

EVALI is a serious pulmonary disease with public health implications. Diagnosis requires a high degree of suspicion to diagnose and exclusion of other possible causes of lung disease. It may be beneficial to involve a pulmonary specialist early in the management of this disease which is generally supportive care.

## Background

Electronic cigarettes (e-cigarettes) are known by many different names, including e-cigs, mods, vapes, and electronic nicotine delivery systems [1]. E-cigarettes are battery-operated devices that produce an aerosol by heating substances typically containing nicotine and/or other materials or flavorings on an internal metal coil until the material is aerosolized and can be inhaled [2, 3]. “Vaping” is a broad term which denotes inhaling the aerosol produced by an e-cigarette or other vaporizing device. E-cigarettes began to appear in US markets in 2006, and enjoyed a growing demand among youth and adults, culminating with the US Surgeon General naming e-cigarette use a growing public health “epidemic” [4]. Since entering the US marketplace, several generations of e-cigarette products have been developed with newer versions that can deliver higher concentrations of aerosol [5, 6]. Over 7000 flavors and other chemical constituents have been identified within the e-cigarette makeup, including some with carcinogenic potential [7–9]. Outside of nicotine-based use, e-cigarettes have also become popular as a mode of tetrahydrocannabinol (THC) delivery.

Since the introduction of these devices, it has been postulated that inhalation of microparticles and volatile chemicals produced by the vaping process could injure the lungs. Isolated reports of lung injury considered to be due to vaping began in 2012 [10–14]. Then, in July, 2019, the Illinois Department of Public Health (IDPH) and Wisconsin Department of Health Services (WDHS) began a joint public health investigation after receiving reports of clusters of cases of lung injury that occurred after vaping. The New England Journal of Medicine published the first series of 142 reported cases of “Pulmonary Illness Related to E-cigarette use in Illinois and Wisconsin” from this collaboration [15]. This disease was ultimately termed “EVALI “(E-cigarette, or Vaping, Product Use-Associated Lung Injury). Since then there has been a rapid increase in reporting of this disease to public health authorities peaking in September of 2019, accompanied by policy efforts to restrict the distribution of these devices and materials. As of February, 2020, there have been 2668 hospitalized EVALI cases reported to the Centers for Disease Control and Prevention (CDC) [16, 17]. In this article we will review the epidemiology, pathogenesis, and clinical features important to managing patients with suspected EVALI.

## Epidemiology and demographics

Precise data concerning the epidemiology of vaping and its associated lung injury are difficult to confirm. Information is often self-reported by patients and/or family and is subject to recall and willingness to report various and possibly illegal activities, such as THC use – a Federal crime despite being legal in many states. It is known that vaping has been gaining immense popularity among young people and e-cigarettes are the most commonly used tobacco product among youth [4]. In 2019 over 5 million children and adolescents were using e-cigarettes. This represented an increase in e-cigarette use by high school students from 11.7% in 2017 to 27.5% in 2019 [18].

Since being recognized in the summer of 2019 as a serious pulmonary disease with public health implications more than 2600 cases have been reported to CDC with 68 deaths as of February 2020 [17]. Among these cases, 66% were male and approximately 76% were under the age of 35 years. Given the age distribution and rapid increase in e cigarette use by young people, it is not surprising that the reports of EVALI are primarily among adolescents and young adults. A large portion of these patients reported using THC-containing products in conjunction with nicotine-containing products [17]. It is important to note that while sporadic reports of severe lung disease associated with using e-cigarette products have been reported since 2012 [10–14], patients from these earlier reports less frequently reported cannabis use, unlike the 2019 outbreak [10].

## Pathogenesis/etiology and risk factors

EVALI is a form of acute lung injury with varying pathologic findings, ranging from acute fibrinous pneumonitis to organizing pneumonia to diffuse alveolar damage [19]. In conjunction with these histopathologic findings, cases of EVALI have presented as acute eosinophilic pneumonia, lipoid pneumonia (although this diagnosis is controversial and will be addressed later in this review), and respiratory-bronchiolitis interstitial lung disease (RB-ILD) [13, 20, 21]. Differences in clinical and radiographic appearances may be due to a variety of factors, such as underlying lung disease, individual variations in host responses to the inhaled substance, and the specific material inhaled, which is often difficult to determine. Hence, a universal, single etiology has not been determined.

The essential identifiable risk factor for development of EVALI is the use of an e-cigarette or similar device [1]. E cigarette products and aerosols may contain tobacco-specific nitrosamines, aldehydes, metals, volatile organic compounds, phenolic compounds, polycyclic aromatic hydrocarbons, tobacco alkaloids, flavorings and drugs. For example, there is substantial evidence showing that propylene glycol, vitamin E acetate, and metals such as lead and arsenic are important components of some e-cigarettes [22–24]. Propylene glycol and glycerol are typically used as diluents in nicotine-containing e-cigarette products, whereas oils (e.g. medium chain triglycerides) are often used as diluents in THC products [25]. In a murine model, inhalation e-cigarette vapor containing propylene glycol and glycerol has been shown to impair lipid homeostasis and host immune defense [26].

Specific to the outbreak of EVALI in 2019, investigations have shown evidence of THC and/or vitamin E acetate in the majority of affected patients, either by history or confirmed by toxicology [15, 19, 27–30]. Further, vitamin E acetate was widely used as a diluent in THC-containing e-cigarettes between 2018 and 2019 [31]. Vitamin E acetate may alter lung surfactant function and cause respiratory impairment [32]. Heating vitamin E acetate may also generate ketene, a highly reactive compound that acts as a lung irritant [29, 33]. While vitamin E acetate may be a major causative agent, other constituents are likely playing a role as well, including cannabinoid (CBD) oils, petroleum distillates, and limonene [27–29]. Another important risk factor that has been identified includes the source of the material that is vaporized. Studies have shown that a large portion of EVALI cases have been associated with the use of e-cigarettes purchased from an illicit or informal distributer [17, 28]. Additionally, the practice of super-heating the liquid by “dripping” and “dabbing” it onto a torch-flamed spike (nail) using specific devices designed for inhalation of the concentrated vapors produced by the procedure may also lead to production of toxic new agents [27].

## Diagnosis

EVALI should be suspected in patients with a history of vaping within 90 days, a pneumonia-like illness, progressive dyspnea, and/or worsening hypoxemia. CDC has established case definitions of “confirmed EVALI” cases to help guide identification of the disease. The criteria used for a case definition of EVALI (See Table 1) include 1) use of an e-cigarette or related product within 90 days, 2) lung opacities on chest imaging, 3) exclusion of lung infection including viral polymerase chain reaction, basic urine antigen tests for *Legionella* and *Streptococcus pneumoniae*, blood cultures, and sputum culture, and 4) absence of a likely alternative diagnosis such as cardiac or neoplastic conditions [34]. Despite the established CDC criteria to assist in identifying EVALI cases, there are a variety of respiratory diseases that may present similarly or even in association with EVALI [15, 19]. The differential diagnosis, outside of community acquired pneumonia (CAP) and viral pneumonia, includes parenchymal lung diseases such as acute eosinophilic pneumonia, organizing pneumonia, hypersensitivity pneumonia, lipoid pneumonia, diffuse alveolar hemorrhage, giant cell pneumonitis, and RB-ILD, and cardiac causes such as congestive heart failure [11, 13, 15, 20, 21, 35–40].

**Table 1:** CDC Surveillance Case Definitions^a^ for Severe Pulmonary Disease Associated with Cigarette Use – August 30th 2019 [34]

Case Classification	CDC Criteria	Additional investigations to consider:
**Confirmed**	Using an e-cigarette (“vaping”) or dabbing^b^ during the 90 days before symptom onset (and)	Consider toxicology to assess for THC or other inhalational agents
	Pulmonary infiltrate, such as opacities on plain film chest radiograph or ground-glass opacities on chest computed tomography (and)	Consider CT scan for increased sensitivity
	Absence of pulmonary infection on initial work-up: Minimum criteria include negative respiratory viral panel, influenza polymerase chain reaction or rapid test if local epidemiology supports testing. All other clinically indicated respiratory infectious disease testing (e.g., urine antigen for *Streptococcus pneumoniae* and *Legionella*, sputum culture if productive cough, bronchoalveolar lavage culture if done, blood culture, human immunodeficiency virus–related opportunistic respiratory infections if appropriate) must be negative (and)	HIV testing SARS-CoV-2 testing Procalcitonin CBC with differential
	No evidence in medical record of alternative plausible diagnoses (e.g., cardiac, rheumatologic, or neoplastic process).	Echocardiography Differential on CBC ANA RF ANCA ESR CRP
**Probable**	Using an e-cigarette (“vaping”) or dabbing^b^ in 90 days before symptom onset (and)	Consider toxicology to assess for THC or other inhalational agents
	Pulmonary infiltrate, such as opacities on plain film chest radiograph or ground-glass opacities on chest computed tomography (and)	Consider CT scan for increased sensitivity
	Infection identified via culture or polymerase chain reaction, but clinical team^c^ believes this is not the sole cause of the underlying respiratory disease process **OR** minimum criteria to rule out pulmonary infection not met (testing not performed) and clinical team^c^ believes this is not the sole cause of the underlying respiratory disease process (and)	HIV testing SARS-CoV-2 testing Procalcitonin CBC with differential
	No evidence in medical record of alternative plausible diagnoses (e.g., cardiac, rheumatologic, or neoplastic process).	Echocardiography Differential on CBC ANA RF ANCA ESR CRP

^a^These surveillance case definitions are meant for surveillance and not clinical diagnosis; they are subject to change and will be updated as additional information becomes available if needed

^b^Using an electronic device (e.g., electronic nicotine delivery system (ENDS), electronic cigarette (e-cigarette), vaporizer, vape(s), vape pen, dab pen, or other device) or dabbing to inhale substances (e.g., nicotine, marijuana, tetrahydrocannabinol, tetrahydrocannabinol concentrates, cannabinoids, synthetic cannabinoids, flavorings, or other substances)

^c^Clinical team caring for the patient

## Clinical presentation and evaluation

EVALI remains a clinical diagnosis and one of exclusion as the symptoms, physical examination, serologic, radiologic, and bronchoscopy findings are not specific to the disease. While clinical judgement by healthcare providers is imperative in the identification of EVALI cases, the heterogeneous presentations of EVALI make this difficult as well. Additionally, bacterial or viral co-infection can occur. This is particularly important to acknowledge during flu-season or in the era of COVID-19, both of which can present similarly or even concurrently with EVALI.

### History

Patients with EVALI may present with respiratory symptoms, constitutional symptoms, and/or gastrointestinal symptoms and assessments for these symptoms are imperative when evaluating patients suspected of EVALI. As of October 2019, 342 EVALI patients with medical abstraction data were submitted to CDC for chart review, 3 of which were excluded due to incomplete data. Respiratory symptoms (including cough, chest pain, shortness of breath) were reported in 95% (323/339) of patients; 85% (289/339) reported constitutional symptoms (including weight loss, fevers, chills) and 77% (262/339) had gastrointestinal symptoms (including nausea, vomiting, diarrhea, abdominal pain) [41]. Similar numbers were reported by the Illinois/Wisconsin Cohort in which 142 patients were submitted for review 30 of whom were excluded after chart review and 14 were excluded due to pending classification leaving 98 to be evaluated. Of these patients, reported symptoms included shortness of breath in 85% (83/98) patients, cough in 85% (83/98), chest pain in 52% (51/98), pleuritic chest pain in 36% (35/98), hemoptysis in 8% (8/98), fevers in 84% (82/98), chills in 60% (59/98), and gastrointestinal symptoms in 77% (75/98) [15]. It is also important when taking the history to assess other potential causes of the patient’s illness such as infectious, cardiac, autoimmune, or inflammatory disorders, as part of the diagnosis of EVALI is ruling out alternative diagnoses.

Additionally, there are specific components to the evaluation of a patient with suspected EVALI. Non-judgmental, open-ended, and private questioning should be used in order to obtain an accurate history. This is particularly important in the adolescent population [42]. Some specific details related to substance use should be asked including: start date, last use, method of use (aerosol, dabbing or dripping), duration of use, frequency of puffs, and concomitant tobacco or other drug use. Additionally, details regarding the actual device should be obtained including: product brand name, delivery system, types of substances used for vaping (THC, cannabis, nicotine, modified products), and the product source [43].

### Physical examination

In patients diagnosed with EVALI reported to CDC, tachycardia, tachypnea and oxygen saturation < 95% have been documented in 55% (169/310), 45%(77/172) and 57% (143/253) of cases respectively. Denominators are different for selected characteristics to account for exclusion of patients with missing data [41]. In the Illinois/Wisconsin Cohort, fever was recorded in the vital signs in 33% of patients [15]. The physical exam should target the cardiopulmonary system including vital signs and pulse oximetry, not only to assess for severity of respiratory distress, but also to assess for other etiologies of respiratory illness such as chronic lung disease, congestive heart failure or community acquired pneumonia.

### Laboratory testing

Because EVALI is a diagnosis of exclusion, lab testing should focus on ruling out alternative diagnoses. Viral respiratory panel testing should be considered as well as specific influenza A and B testing during flu season [41]. Additionally, testing of infectious disease including but not limited to *Streptococcus pneumonia*, *Legionella pneumophila*, fungal infections, HIV, COVID-19 and opportunistic infections should be considered. Case reports and case series including the Illinois/Wisconsin Cohort have documented elevation in inflammatory markers such as C-reactive protein, erythrocyte sedimentation rate and white blood cell count [15]. The CDC, in its Morbidity and Mortality Weekly Report in October 2019, noted reports of elevated inflammatory markers in EVALI patients, but commented that these laboratory findings remain non-specific and may not be particularly helpful in ruling out other etiologies [41]. In order to evaluate etiologies of lung diseases precipitated by other illicit substances, toxicology testing should be considered with appropriate consenting [41].

### Imaging

**Fig. 1:**
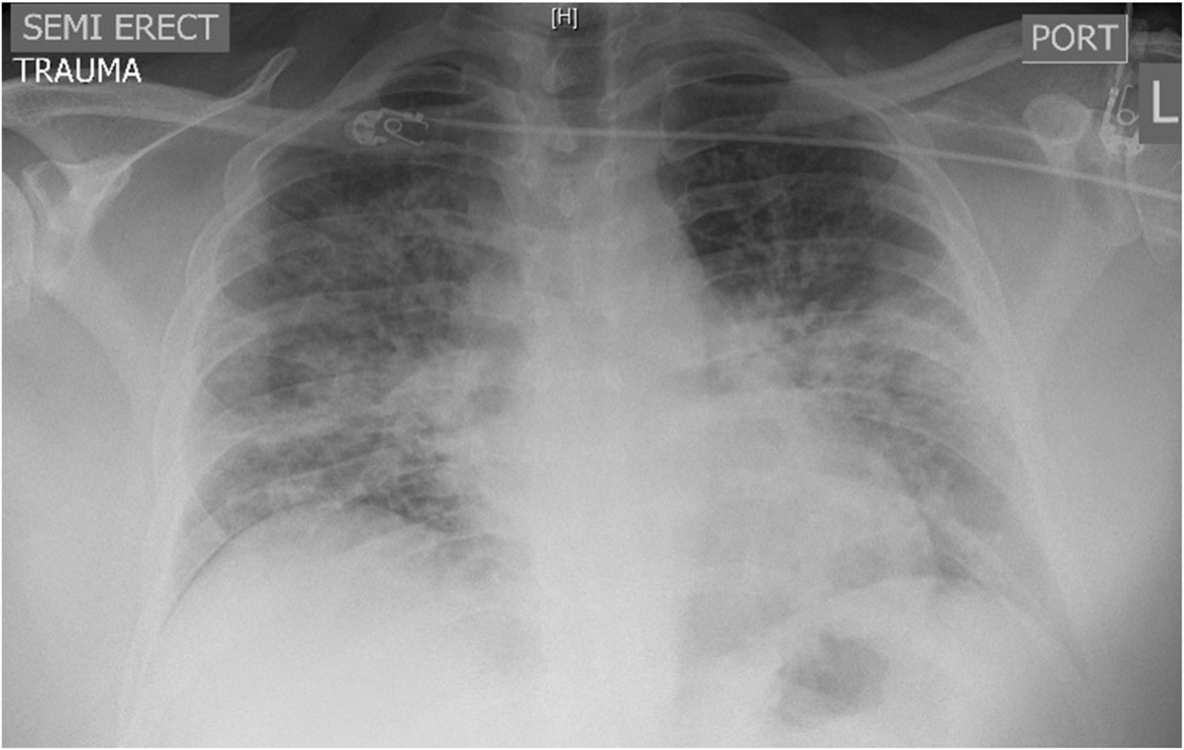
Chest XRay of a patient with EVALI Showing bilateral patchy opacities

**Fig. 2:**
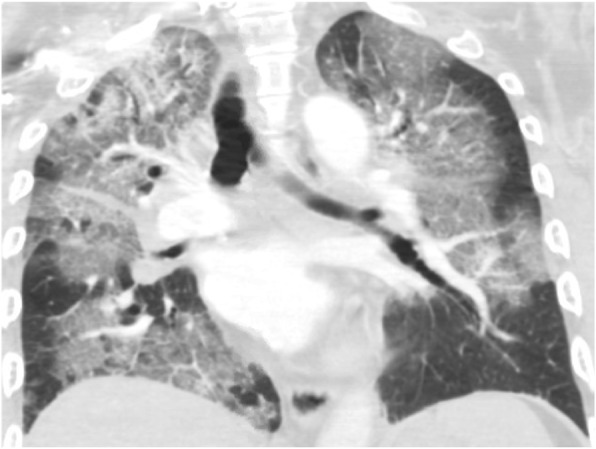
Computed Tomography coronal image through the chest of a patient wih EVALI showing bilateraly patchy ground glass opacities

Although abnormalities are frequently found on chest imaging, the findings are non-specific and variable. In the Illinois/Wisconsin cohort, 83% were found to have abnormalities on chest radiograph and 100% were found to have abnormalities on Computed Tomography (CT) of the chest [15]. A chest x-ray should be obtained in patients with e-cigarettes use who present with respiratory, GI, or constitutional symptoms. Typical of findings on chest radiograph of EVALI is diffuse hazy bilateral opacities with occasional subpleural sparing (Fig. 1). Involvement of all lung lobes can be seen, but is not universal. Additionally, increased interstitial markings can be seen characterized by Kerley B lines. A CT chest should be pursued if there is high suspicion for EVALI but the chest radiograph is normal given the improved sensitivity of CT and/or to assist in ruling out other etiologies. While imaging findings are variable in EVALI, typical findings on chest CT are bilateral ground glass opacities (Fig. 2). Additionally, upper lobe predominant centrilobular nodules are often seen on chest CT [44]. Since findings on chest imaging are non-specific, other etiologies of lung injury should be considered.

### Bronchoscopy

Bronchoscopy has been used both to obtain bronchoalveolar lavage (BAL) and biopsy specimens. Cellular analysis of BAL specimens are of little diagnostic utility since there is no specific cellular pattern in EVALI. A common finding in BAL specimens in patients with EVALI is lipid-laden macrophages [19, 30, 31]. This finding initially prompted concern that this was a disease of lipoid pneumonia. However, radiographic features are not typical of lipoid pneumonia. Additionally in multiple series, biopsy specimens revealed foamy (lipid-laden) macrophages within airspaces but did not have features consistent with lipoid pneumonia such as coarse vacuolation or giant cells of lipoid pneumonia [19, 45]. Lipid-laden macrophages may therefore instead represent an endogenous response to e-cigarettes.

CDC also has reported finding vitamin E acetate in the BAL specimens submitted from 29 of 29 patients from 10 states [30]. A follow-up study of 51 patients found that 94% (48/51) of patients with EVALI had detectable vitamin E acetate in BAL with no detectable vitamin E acetate in the BALs of the healthy control group. While not firmly established as the universal cause of this injury, Vitamin E acetate found in lavage fluids strengthened evidence considerably. However multiple factors could potentially limit the value of negative results such as: time elapsed between last use of E-cigarette and bronchoscopy, variations in bronchoalveolar lavage technique and uncontrolled dilution of alveolar fluid by instilled saline [46]. Additionally, vitamin E acetate testing is not performed routinely by many laboratories.

In a case series of 8 biopsy specimens (7 of which were obtained by transbronchial biopsy via bronchoscopy, 1 by open surgical lung biopsy) from patients with EVALI, pathology revealed a mix of organizing pneumonia and diffuse alveolar damage [47]. In a case series submitted to the New England Journal of Medicine, all 17 cases showed a combination of acute lung injury in nonspecific patterns including fibrinous pneumonitis, diffuse alveolar damage, and organizing pneumonia [19]. These are all patterns of injury which can result from multiple different insults. Since there are no specific findings on biopsy for EVALI, routine biopsy for confirmatory testing of EVALI is generally not recommended.

Given that EVALI remains a diagnosis of exclusion, bronchoscopy can help evaluate and rule out alternative or concomitant diagnoses such as infection, malignancy, or eosinophilic pneumonia. The decision to pursue a bronchoscopy is made on a case-by-case basis and should be made by the clinical team in consultation with pulmonary specialists.

## Treatment

The approach to treatment for EVALI is focused primarily on elimination of the insult and supportive therapy. Outpatient management can be considered in patients with S_a_O_2_ > 95% on room air. The CDC recommends that patients managed in the outpatient setting should have reliable access to care and social support systems so as to ensure follow-up within 24–48 h to assess for worsening lung injury [41]. Patients should also be provided instructions to seek prompt medical care if respiratory symptoms worsen. The CDC recommends hospital admission for patients how have decreased oxyhemoglobin saturation (S_a_O_2_ < 95%) on RA who or who are in respiratory distress.

At present, there is no optimal treatment regimen for EVALI. Vaping must cease. The supportive care focuses on supplemental oxygenation with a target pulse oxygen saturation of 88 to 92%; this can be achieved through high flow nasal cannula if nasal cannula, alone, is insufficient. The approach to managing oxygenation in EVALI cases mirrors that of treatment algorithms of acute respiratory distress syndrome (ARDS) since 26% of patients in one cohort required mechanical ventilation [15]. Similar to its function as rescue therapy for severe ARDS not responding to ARDS ventilator management, venovenous extracorporeal membranous oxygenation (VV-ECMO) has been used successfully in cases reports of EVALI [40, 48, 49].

Empiric antibiotics are often initiated to cover likely pathogens of CAP as well as antivirals during influenza season. Antibiotics may be continued during the initial evaluation and if a concomitant infection eventually is ruled out, they can be discontinued. Along with antibiotics, systemic glucocorticoids have also been utilized as adjunct therapy in the majority of EVALI patients [15, 37, 40].

Observational studies have shown clinical improvement in response to corticosteroids, but it is unclear whether clinical improvement was due to steroids since the natural history of untreated EVALI is not known [15, 37, 40]. A retrospective chart review of pediatric EVALI cases at single hospital revealed improvement in multiple pulmonary function testing (PFT) parameters (including forced expiratory volume in 1 s, forced vital capacity, total lung capacity and diffusion capacity of carbon monoxide) in all patients who received steroids and had PFTs performed, however there was no control group [50]. Ultimately, each patient must be evaluated on a case-by-case basis to determine if the benefits of glucocorticoid therapy outweigh the risks [19, 51].

Additional experimental therapeutic options for the treatment of EVALI are being evaluated. Scott et al. reported that e-cigarette vapor condensate is significantly more toxic to alveolar macrophages when compared to e-cigarette fluid that is not vaporized [52]. In this in-vitro study, the investigators showed that using the anti-oxidant N-acetylcysteine (NAC) can significantly attenuate the cytotoxic and pro-apoptotic effects of the e-cigarette fluid vapor condensate. Choe et al. reported a case of EVALI with favorable outcomes following treatment with inhaled NAC that was being used for its mucolytic properties [53]. While there are theoretical benefits for using NAC in patients with EVALI, further investigation into the therapeutic role of NAC in EVALI management is needed.

## Discharge and follow-up

Prior to discharge from the hospital, it is imperative to ensure that the patients’ subjective dyspnea has resolved and that vital signs including oxygenation have stabilized for 24 to 48 h. There is little known about whether resuming vaping after an EVALI diagnosis increases risk for recurrent disease. In a multicenter, prospective, observational study on EVALI patients seen in an integrated health system in Utah, USA (June 27 and Oct 4, 2019), 6 (10%) of 60 patients were readmitted to the ICU or hospital within 2 weeks, of which three (50%) had relapsed with e-cigarette use [54]. Since neither the risk factors for reoccurrence nor the exact mechanisms of EVALI are known, recommending that EVALI patients completely stop vaping and providing appropriate cessation counseling should be an integral part of discharge care. Lastly, those admitted with comorbid conditions may require close follow-up after discharge, since re-hospitalization and post-discharge mortality may be high in those of older age and with underlying chronic conditions [55]. The CDC updated their recommendation for follow-up after hospital discharge from 2 weeks to just 2 days.

Outside of the short-term complications following a diagnosis of EVALI, much is unknown about the long-term sequela of the disease process. Therefore, follow-up evaluation with a Pulmonology specialist as well as addiction counseling may be warranted and establishing a multi-disciplinary program to provide comprehensive care for EVALI patients, as well as routine subjective and objective monitoring, is crucial [56].

## Reporting

EVALI is considered a reportable illness in some states and not others. This information can be found at the websites of individual state health departments to which CDC provides a directory and hyperlink [57]. Details on laboratory collection and specimen submission can also be found on the CDC website [58].

## Prevention

The CDC recommends avoidance of all THC-containing e-cigarette and vaping products as a way to prevent EVALI [17]. Multiple policy initiatives have attempted to minimize and regulate vaping. Most prominently, the passage of the Tobacco 21 legislation in November 2019, that was signed into law December 2019, increased the minimum age of sales from 18 years to 21 years nationwide [59]. Additionally, there have been attempts to combat the sale of flavored nicotine products in order to reduce the appeal to younger people. Effective February 2020, the FDA banned flavored cartridge-based e-cigarette products, except menthol and tobacco flavorings. Certain products, however, do not apply to this ban such as flavorings for non-pod devices. Effective e-cigarette control policy should strongly consider a complete ban on all flavored e-cigarette products, restricting online sales, and taxing e-cigarette/vaping products to decrease youth initiation.

## Conclusions

EVALI is a serious pulmonary disease with public health implications. The diagnostic evaluation of patients with suspected EVALI remains focused on ruling out alternative and concomitant diagnoses as EVALI remains a diagnosis of exclusion. This should frequently be done in conjunction with pulmonary specialists familiar with the disease. Chest imaging is relatively sensitive for EVALI but the findings are non-specific. Bronchoscopy is most useful to help in rule out other diagnoses. All patients diagnosed with EVALI should be instructed to abstain from using e-cigarettes or other vaping products in the future. Outpatient follow-up with a pulmonary specialist should be considered because little is known about the long-term sequelae of this disease.

## Data Availability

See references.

## Abbreviations

EVALI: E-cigarette, or Vaping, Product Use-Associated Lung Injury
CDC: Centers for Disease Control and Prevention
THC: Tetrahydrocannabinol
IDPH: Illinois Department of Public Health
WDHS: Wisconsin Department of Health Services
RB-ILD: respiratory-bronchiolitis interstitial lung disease
CT: Computed Tomography
FDA: Food and Drug Administration
